# The association of moderate renal dysfunction with impaired preference-based health-related quality of life: 3^rd ^Korean national health and nutritional examination survey

**DOI:** 10.1186/1471-2369-13-19

**Published:** 2012-04-24

**Authors:** Hajeong Lee, Yun Jung Oh, Myounghee Kim, Ho Kim, Jung Pyo Lee, Sejoong Kim, Kook-Hwan Oh, Ho Jun Chin, Kwon Wook Joo, Chun Soo Lim, Suhnggwon Kim, Yon Su Kim, Dong Ki Kim

**Affiliations:** 1 Department of Internal Medicine, Seoul National University Hospital, Seoul, South Korea; 2 Department of Epidemiology and Biostatistics, School of Public Health, Seoul National University Hospital, Seoul, South Korea; 3 Department of Internal Medicine, Seoul National University Boramae Medical Center, Seoul, South Korea; 4 Department of Internal Medicine, Seoul National University Bundang Hospital, Seongnam, South Korea; 5 Kidney Research Institute, Seoul National University Hospital, Seoul, South Korea; 6 Department of Internal Medicine, Seoul National University Hospital, 101 Daehakro, Jongro-gu, 110-744 Seoul, Republic of Korea

**Keywords:** Chronic kidney disease, EuroQol-5D, Preference-based health utility

## Abstract

**Background:**

Only a few large-scale studies have investigated the association between health-related quality of life (HRQOL) and renal function. Moreover, the HRQOL of patients with moderate renal dysfunction is frequently underestimated by healthcare providers. This study assessed the impact of renal function on preference-based HRQOL in Korean adult population.

**Methods:**

We analyzed data for 5,555 adults from the 3^rd ^Korean National Health and Nutritional Examination Survey 2005. The EuroQol-5D (EQ-5D) utility score was used to evaluate HRQOL. The study subjects were stratified into three groups based on their estimated glomerular filtration rates (eGFRs): ≥ 90.0, 60.0-89.9 and 30.0-59.9 mL/min/1.73 m^2^. Individuals with advanced renal dysfunction were excluded from the analysis.

**Results:**

The proportions of participants who reported problems in each of the five EQ-5D dimensions increased significantly with decreasing eGFR. However, a significant decrease in the EQ-5D utility score was observed among participants with an eGFR of 30.0-59.9 mL/min/1.73 m^2^. Participants with an eGFR of 30.0-59.9 mL/min/1.73 m^2 ^had an almost 1.5-fold higher risk of impaired health utility (the lowest quartile of EQ-5D utility score) compared with those participants with eGFRs ≥ 90.0 mL/min/1.73 m^2^, after adjustment for age, gender, health-related behaviors, socioeconomic and psychological variables, and other comorbidities. Among the five dimensions of the EQ-5D, an eGFR of 30.0-59.9 mL/min/1.73 m^2 ^was an independent determinant of self-reported problems in the mobility and pain/discomfort dimensions.

**Conclusions:**

Although age affects the association between renal dysfunction and the EQ-5D, moderate renal dysfunction seems to be an important determinant of impaired health utility in a general population and may affect the mobility and pain/discomfort dimensions of health utility.

## Background

Generic preference-based health-related quality of life (HRQOL) instruments, which generate health state values as a single numerical index, have been proposed for use in health-economic analyses for comparing HRQOL across different diseases and allocating proper healthcare resources [1,2]. Because chronic kidney disease (CKD) is highly interactive with various comorbidities including diabetes, cardiovascular and cerebrovascular diseases, the substantial healthcare expenditure for patients with CKD cannot be directly attributed to CKD itself [3]. Indeed, an inadequate financial policy for pre-dialysis CKD is one of the barriers preventing improved patients outcomes [4]. Thus, preference-based HRQOL measurement based on renal function is needed for proper allocation of healthcare resources to CKD patients.

Although CKD is a progressive and life-long condition with multiple medical comorbidities, its implications for HRQOL have only been studied, for the most part, in the advanced stages of CKD (stage 4-5) [5]. The HRQOL in patients with advanced CKD is significantly impaired and is an important indicator of future mortality [6]. More recently, however, the high mortality rate and prevalence of comorbid conditions even in patients in the earlier stages of CKD [7] has raised concerns that HRQOL could also be reduced in these patients. Indeed, there is considerable evidence of decreased HRQOL among patients with mild-to-moderate renal dysfunction compared with the population with normal renal function [8-11]. However, only limited information is currently available regarding estimates of the relative impact of renal dysfunction on HRQOL and predictors of HRQOL as targets of intervention. Previous studies have demonstrated that various comorbid conditions, such as anemia, hypertension, frailty, symptom burden, and depression, negatively affect HRQOL in pre-dialysis CKD patients [12]. However, these studies have limited generalizability because they either are based on a non-representative sample [9,13-16] or do not allow for integration of HRQOL measures into health-economic analyses because of the use of non-preference-based models [11].

Therefore, a population-based study of the relationship between renal function and preference-based health utility measures may contribute to a comprehensive public health strategy for the management of CKD. In the present study, we analyzed population-based data from a nationwide cross-sectional health survey to determine the association of health utility with mild-to-moderate renal dysfunction.

## Methods

### Participants

The data analyzed in this study were obtained from the 3^rd ^Korean National Health and Nutritional Examination Survey (KNHANES) 2005, which included a population-based random sampling of 34,145 individuals in households across 600 national districts. The survey was conducted with a stratified, multi-stage, clustered probability design in order to select a representative nationwide sample of the non-institutionalized Korean population. A total of 28,590 subjects were excluded from this study because they were age < 18 years (n = 8,292), did not complete either the EQ-5D questionnaire (n = 9,720) or blood test (n = 27,731), or had advanced renal dysfunction (n = 12). After the above exclusion criteria were applied, 5,555 individuals aged 18 years or older who had an estimated glomerular filtration rate (eGFR) ≥ 30 mL/min/1.73 m^2 ^were included in this investigation. Because the analyzed survey data are publicly available, ethical approval was not required for this study.

### Health-related quality of life

HRQOL was measured using the EuroQol-5D (EQ-5D) questionnaire, a widely used generic preference-based instrument [17,18]. The EQ-5D consists of five questions regarding current health status in terms of mobility, self-care, usual activities, pain or discomfort, and anxiety or depression. Each question has three possible responses: "no problems", "some problems", and "extreme problems". The EQ-5D health states are defined as a combination of the responses for each item and the survey can therefore yield 3^5 ^(= 243) possible combinations of responses. These responses were converted into weighted values according to the Korean value set [19], and the average was calculated as a quality adjustment weight for each health state. The EQ-5D instrument has been translated into Korean, and its validity (Spearman correlation coefficient with the first question of the Health Survey Short-Form 36:-0.51 in EQ-5D) and reliability (test-retest reliability) have been demonstrated previously [19,20].

### Laboratory parameters

Blood samples were collected after a 12-hour overnight fast; they were properly processed, immediately refrigerated, and transported in cold storage to the central laboratory (Seoul Medical Science Institute, Seoul, Korea) within 24 hours. Serum creatinine, glucose, and lipid levels were measured using the ADVIA 1650 system (Bayer Health Care, Tarrytown, NY). The serum creatinine concentration was measured using the kinetic Jaffe method, and the inter-assay coefficient of variation was less than 5%. Because the creatinine assay was not calibrated to be traceable to an isotope dilution mass spectrometry (IDMS), eGFR was calculated using the original Modification of Diet in Renal Disease (MDRD) equation as follows: eGFR = 186.3 × (serum creatinine)^-1.154 ^× (age)^-0.203 ^× 0.742 (if female) [21]. Proteinuria was measured by the urine dipstick test.

### Demographic and clinical characteristics

Demographic characteristics included age, gender, marital status (living with/without a spouse), education level (no education or elementary school graduate/middle or high school graduate/university graduate or higher), occupational status (white collar/blue collar/student, soldier or housewife/no occupation), residential area (rural/urban), and monthly individual income (lowest quartile/2^nd ^and 3^rd ^quartile/highest quartile) in US dollars. Individuals who were legally married or cohabiting were considered to have a spouse; single, divorced, or separated individuals were categorized as not having a spouse. Information about various comorbidities was also collected. Hypertension was identified in individuals who met at least one of the following three criteria: physician diagnosis of hypertension, self-report of antihypertensive drug intake, and systolic blood pressure (SBP) ≥ 140 mmHg or diastolic blood pressure (DBP) ≥ 90 mmHg. Blood pressure was measured manually twice at 30-second intervals after a minimum of five minutes of rest in a seated position, and the mean values were used to identify hypertensive participants. Diabetes was diagnosed in subjects with a fasting plasma glucose ≥ 126 mg/dL or those patients who were identified in the health interview survey as actively using an oral hypoglycemic agent or insulin. Diagnosis of metabolic syndrome was based on the presence of three or more of the following: (1) waist circumference ≥ 90 cm for men or ≥ 80 cm for women [22], (2) triglyceride levels ≥ 150 mg/dL, (3) high-density lipoprotein cholesterol levels < 40 mg/dL for men or < 50 mg/dL for women, (4) SBP ≥ 130 mmHg or DBP ≥ 85 mmHg or self-report of antihypertensive drug therapy, and (5) fasting plasma glucose level ≥ 100 mg/dL or self-report of ongoing treatment with an oral hypoglycemic agent or insulin. Anemia was defined as a hemoglobin level of < 13 g/dL for men and < 12 g/dL for women. Information regarding ischemic heart disease and cerebrovascular accidents was acquired from self-reported history. Ischemic heart disease included angina pectoris and myocardial infarction. Proteinuria was categorized into 3 groups according to the degree of proteinuria measured by the dipstick as negative, mild (trace to 1+), or heavy (2+ to 4+).

Information on health-related behaviors such as smoking status (life-time smoker/non-smoker), alcohol intake (less than once per month/more than once per month), and regular physical activity of moderate intensity (more/less than three times per week) was obtained from the health questionnaire. Life-time smokers included those adults who reported that they had smoked at least 100 cigarettes in their lifetime, and non-smokers included respondents who had smoked fewer than 100 cigarettes in their lifetime and did not smoke at the time of the survey. Moderate-intensity activities were defined as those lasting at least 10 minutes and causing a slight increase in the individual's heart rate compared with sedentary activities; table tennis, swimming, yoga and badminton were included as moderate-intensity activities, but walking was excluded. Psychological variables from questionnaires included self-reported stress (none or small amount/some or extreme) and sleep quality (sufficient/insufficient).

### Statistical analysis

Data are presented as frequencies and percentages for categorical variables. Continuous variables are reported as means with standard deviations. Estimated GFR values were stratified into three categories (≥ 90.0, 60.0-89.9, and 30.0-59.9 mL/min/1.73 m^2^). Differences in demographic, socioeconomic, and psychological factors, as well as in health-related behavioral patterns and the EQ-5D utility scores across the eGFR categories were compared using the χ^2 ^test for trends (linear-by-linear association) for categorical variables. Similarly, a one-way analysis of variance was used to demonstrate the linearity of continuous variables across eGFR categories.

Univariate logistic regression analysis was performed to assess the relationship between impaired health utility (EQ-5D index score in lowest quartile) [23] and clinical or demographic data. Variables that showed significant association in the univariate analysis or that were of considerable theoretical relevance were entered into the multivariate logistic regression analysis using the backward conditional elimination method. To analyze the determinants of problems in each of the five EQ-5D dimensions, the three possible responses were dichotomized as "no problem" or "any problem", and a multivariate logistic regression analysis was performed with the presence of "any problem" as the dependent variable. Covariables that had co-linearity were excluded from the multivariate analyses. An analysis of covariance (ANCOVA) with the Bonferroni correction was used to estimate age-adjusted distributions of the EQ-5D utility score according to serum eGFR. All analyses were conducted using SPSS software (version 19.0, SPSS, IL), and *P *< 0.05 was considered to indicate statistical significance.

## Results

### Characteristics of the study population

The demographic characteristics of the participants, stratified by eGFR, are shown in Table 1. The mean age of the study subjects was 46.5 ± 15.7 years, and 42.8% were male. A total of 716 participants had an eGFR of ≥ 90.0 mL/min/1.73 m^2^, 4,353 had an eGFR of 60.0-89.9 mL/min/1.73 m^2^, and 486 had an eGFR of 30.0-59.9 mL/min/1.73 m^2^. The mean eGFR of each group was as follows: 96.7 mL/min/1.73 m^2 ^in the group with eGFRs of ≥ 90.0 mL/min/1.73 m^2^; 75.3 mL/min/1.73 m^2 ^in the group with eGFRs of 60.0-89.9 mL/min/1.73 m^2^, and 55.9 mL/min/1.73 m^2 ^in the group with eGFRs of 30.0-59.9 mL/min/1.73 m^2^. Subjects with a lower eGFR were older, predominantly women, and more likely to have comorbidities, including diabetes, hypertension, metabolic syndrome, anemia, ischemic heart disease and cerebrovascular accidents.

**Table 1:** Demographic characteristics of participants by eGFR

		eGFR (mL/min/1.73 m^2^)			
			
	All (n = 5,555)	≥ 90.0 (n = 716)	60.0-89.9 (n = 4,353)	30.0-59.9 (n = 486)	*P *for trend
Age	46.5 ± 15.7	32.3 ± 12.1	46.5 ± 14.0	67.7 ± 9.9	< 0.001
Male	2,379 (100)	456 (19.2)	1,835 (77.1)	88 (3.7)	
Female	3,176 (100)	260 (8.2)	2,518 (79.3)	398 (12.5)	
eGFR	76.4 ± 12.2	96.7 ± 5.6	75.3 ± 8.2	55.9 ± 5.5	< 0.001
Proteinuria (%)					0.141
Negative	94.4	93.3	94.9	91.6	
Mild	4.8	5.9	4.5	6.1	
Heavy	0.8	0.7	0.6	2.3	
Co-morbidities (%)					
Diabetes mellitus	7.6	3.7	7.0	19.0	< 0.001
Hypertension	25.3	11.0	24.1	55.3	< 0.001
Metabolic syndrome	29.4	13.8	28.4	60.0	< 0.001
Anemia	11.2	9.5	11.0	16.0	0.001
Ischemic heart disease	2.2	0.4	2.0	6.0	< 0.001
Cerebrovascular disease	2.2	0.5	1.8	8.2	< 0.001

Data are expressed as the mean ± standard deviation or a percentage. eGFR calculated using the modified MDRD formula [21]. Ischemic heart disease included angina pectoris and myocardial infarction. eGFR, estimated glomerular filtration rate; MDRD, Modification of Diet in Renal Disease.

Table 2 shows the variables associated with health-related behaviors, socioeconomic status, and psychological variables. Subjects with a lower eGFR showed better health-related behavioral patterns, including lower rates of smoking and alcohol consumption. On the other hand, the proportion of subjects living in a rural area, having no occupation, or with less education was significantly higher as the eGFR decreased. In addition, household income also decreased with decreasing eGFR. Although the degree of stress did not differ between eGFR groups, the proportion of participants experiencing poor sleep quality increased significantly with decreasing eGFR and was particularly low in the group with eGFRs of 30.0-59.9 mL/min/1.73 m^2^.

**Table 2:** Socioeconomic status, psychological factors, and health-related behavioral patterns of participants stratified to eGFR

		eGFR (mL/min/1.73 m^2^)			
			
	All (n = 5,555)	≥ 90.0 (n = 716)	60.0-89.9 (n = 4,353)	30.0-59.9 (n = 486)	*P *for trend
Marital status: living without a spouse (%)	28.5	48.6	23.2	46.8	0.001
Occupation (%)					< 0.001
White collar	31.9	34.2	34.0	9.5	
Blue collar	26.6	26.1	27.1	22.1	
Student/soldier/housewife	25.9	30.2	25.2	26.1	
No occupation	15.6	9.5	13.6	42.0	
Education (%)					< 0.001
University or higher	25.3	33.1	26.2	4.9	
Upper secondary level	49.9	58.8	51.6	21.4	
Compulsory education	24.9	8.1	22.2	73.8	
Income (US $)	2,090.9 ± 1,489.0	2,221.5 ± 1,326.5	2,166.5 ± 1,509.5	1,298.9 ± 1280.8	< 0.001
Rural residence (%)	22.7	19.0	21.9	35.4	< 0.001
Some or extreme degree of stress (%)	33.9	33.4	33.8	33.6	0.778
Sleep quality: insufficient sleep (%)	35.6	41.1	35.5	29.0	< 0.001
Physical activity: ≤ 3 times per week (%)	13.4	16.2	13.5	5.8	0.145
Smoking: life-time smoker (%)	37.9	46.2	37.9	26.0	< 0.001
Alcohol intake: ≥ once a month (%)	31.2	39.5	32.1	11.6	< 0.001

Data expressed a as a percentage or the mean ± standard deviation. eGFR was calculated using the modified MDRD formula [21]. Upper secondary level of education included middle and high school graduate. Compulsory education meant elementary school graduate or less. eGFR, estimated glomerular filtration rate; MDRD, Modification of Diet in Renal Disease.

### HRQOL: EQ-5D dimensions and health utility score

**Figure 1:**
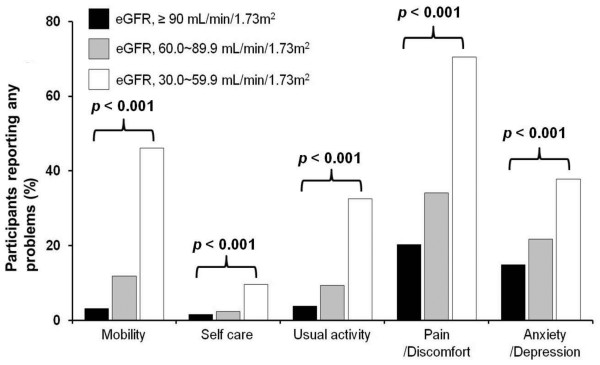
**Proportion of participants who reported problems in each of the five EQ-5D dimensions**.

The proportions of participants reporting problems (some problem/extreme problem) in each dimension of the EQ-5D questionnaire are shown in Figure 1. There was a significant increase in reported problems in all dimensions of the EQ-5D with decreasing eGFR. In total, 46.1% of participants with eGFRs of 30.0-59.9 mL/min/1.73 m^2 ^had problems with mobility, 9.6% had problems with self-care, 32.6% had problems with usual activity, 70.5% had problems with pain/discomfort, and 37.8% had problems with anxiety/depression.

**Figure 2:**
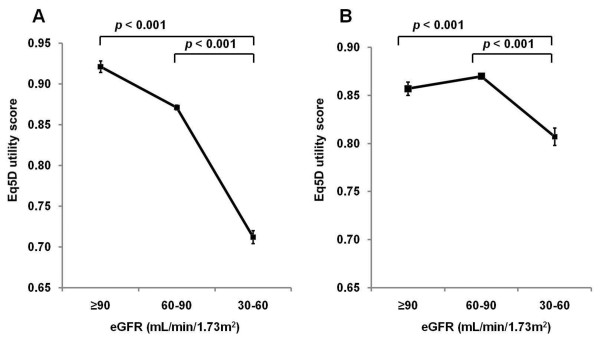
**Mean values of the EQ-5D utility score in a Korean adult population without adjustment (A) and adjusted for age (B)**. Error bars indicate the standard error of the mean.

The crude EQ-5D utility score significantly decreased with decreasing eGFR (Figure 2A). The mean age-adjusted EQ-5D utility score for all participants was 0.845 ± 0.004 (standard error of the mean). The age-adjusted EQ-5D utility score was significantly lower among participants with an eGFR of 30.0-59.9 mL/min/1.73 m^2 ^(0.807 ± 0.009) compared with those participants with an eGFR of ≥ 90.0 mL/min/1.73 m^2 ^(0.857 ± 0.003) or 60.0-89.9 mL/min/1.73 m^2 ^(0.870 ± 0.003) (Figure 2B).

**Figure 3:**
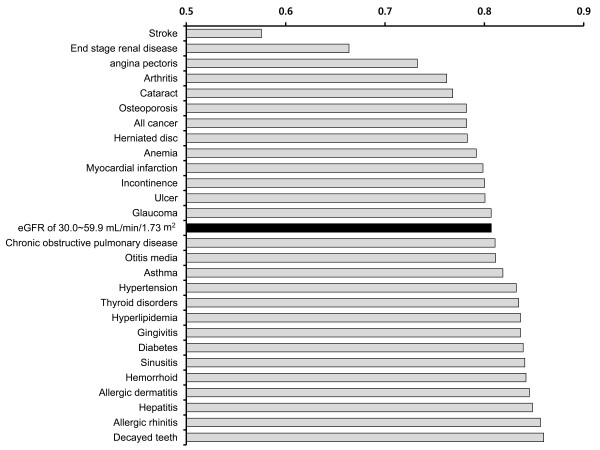
**EQ-5D utility scores for chronic diseases in Korea **[24]. The black bar indicates patients with eGFR 30.0-59.9 mL/min/1.73 m^2 ^who were investigated in this study.

Figure 3 shows data from a nationally representative catalogue of age-adjusted mean EQ-5D utility scores for major chronic diseases, as derived from data from the 3^rd ^KNHANES [24]. When our results were integrated with the data from that catalogue, we found that individuals with an eGFR of 30.0-59.9 mL/min/1.73 m^2 ^had lower utility scores than those individuals with chronic obstructive pulmonary disease, asthma, hypertension, or diabetes.

### Correlates of impaired health utility

A logistic regression model was built to assess factors that were significantly associated with impaired health utility, defined as an EQ-5D utility score in the lowest quartile. To perform logistic regression analysis, certain continuous values were transformed into categorical values, as previously described. Subjects were divided into three age groups, as follows: < 40 years old, 40-60 years old, and ≥ 60 years old. Body mass index and lipid levels were considered as components of metabolic syndrome. Table 3 displays the results of univariate and multivariate analyses for impaired HRQOL. In the univariate regression analysis, variables including age, gender, health-related behaviors (smoking, alcohol intake), socioeconomic factors (marital status, area of residence, occupation, education, and household income), psychological factors (stress, sleep quality), and other co-morbidities (e.g., hypertension, diabetes, metabolic syndrome, ischemic heart disease and cerebrovascular disease) showed significant association with impaired health utility. However, the degree of proteinuria failed to prove its association with health utility in the univariate regression analysis.

**Table 3:** Univariate and multivariate logistic regression analyses for impaired HRQOL

		Univariate analysis		Multivariate analysis	
		
		Unadjusted OR (95% CI)	*P*	Adjusted OR (95% CI)	*P*
Age (year)	< 40	Reference		Reference	
	40-60	2.205 (1.910-2.545)	< 0.001	1.598 (1.345-1.879)	< 0.001
	≥ 60	9.573 (8.117-11.289)	< 0.001	3.337 (2.598-4.287)	< 0.001
Male	Male	0.569 (0.508-0.638)	< 0.001	0.569 (0.487-0.666)	< 0.001
eGFR	≥ 90.0	Reference		Reference	
	60.0-89.9	1.994 (1.639-2.426)	< 0.001	1.168 (0.929-1.469)	0.185
	30.0-59.9	10.372 (7.868-13.672)	< 0.001	1.531 (1.077-2.176)	0.018
Diabetes mellitus	Yes	2.140 (1.747-2.622)	< 0.001		
Hypertension	Yes	2.554 (2.252-2.895)	< 0.001	1.231 (1.048-1.445)	0.011
Metabolic syndrome	Yes	2.091 (1.853-2.358)	< 0.001		
Ischemic heart disease	Yes	8.419 (5.184-13.675)	< 0.001	3.730 (2.169-6.415)	< 0.001
Cerebrovascular accident	yes	3.349 (3.505-8.164)	< 0.001	1.971 (1.194-3.256)	0.008
Alcohol intake	≥ 1/week	0.652 (0.576-0.737)	< 0.001		
Smoking status	life-time smoker	0.789 (0.703-0.886)	< 0.001		
Marital status	without spouse	1.290 (1.141-1.458)	< 0.001		
Occupation	white collar	Reference		Reference	
	blue collar	2.545 (2.184-2.965)	< 0.001	1.400 (1.154-1.689)	0.001
	other^†^	1.944 (1.661-2.275	< 0.001	1.328 (1.097-1.608)	0.004
	no occupation	4.953 (4.148-5.914)	< 0.001	1.741 (1.378-2.199)	< 0.001
Education	≥ university	Reference		Reference	
	upper 2ndary^‡^	1.886 (1.608-2.213)	< 0.001	1.294 (1.076-1.555)	0.006
	≤ compulsory^§^	9.607 (8.035-11.488)	<0.001	2.515 (1.970-3.212)	< 0.001
Income	highest quartile	Reference		Reference	
	2^nd ^-3^rd ^quartile	1.432 (1.224-1.674)	< 0.001	1.139 (0.953-1.360)	0.152
	lowest quartile	4.735 (3.986-5.625)	< 0.001	1.676 (1.356-2.072)	< 0.001
Residence	rural residence	2.056 (1.808-2.339)	< 0.001	1.190 (1.011-1.401)	0.036
Stress	some or extreme	1.752 (1.559-1.968)	< 0.001	1.753 (1.525-2.016)	< 0.001
Sleep quality	insufficient	1.276 (1.137-1.432)	< 0.001	1.606 (1.396-1.848)	< 0.001

Values shown are OR (95% CI). Impaired HRQOL was defined as the lowest quartile of EQ-5D weighted values. Potential risk factors in the unadjusted analysis (*P *< 0.05) were included in the adjusted analysis. The multivariate logistic regression analysis model was derived using the backward conditional method. †Students, soldiers and housewives; ‡middle and high school graduates; §elementary school or no education. HRQOL, health-related quality of life; eGFR, estimated glomerular filtration rate (mL/min/1.73 m^2^); OR, odds ratio; CI; confidence interval

In the multivariate analysis, an eGFR of 30.0-59.9 mL/min/1.73 m^2 ^was one of the independent risk factors predicting impaired health utility (odds ratio (OR) 1.531; 95% confidence interval (CI) 1.077-2.176; *P *= 0.018) after adjustment for age, sex, comorbidities (diabetes, hypertension, metabolic syndrome, ischemic heart disease, and cerebrovascular accidents) health-related behaviors (alcohol intake, smoking, and physical activity), socioeconomic factors (marital status, occupation, education, rural residence, and income) and psychological factors (stress and sleep quality).

In the binary multivariate logistic regression on EQ-5D responses, an eGFR of 30.0-59.9 mL/min/1.73 m^2 ^was significantly associated with reported problems in the mobility (OR, 2.192; 95% CI, 1.178-4.077; *P *= 0.013) and pain/discomfort dimensions (OR, 1.574; 95% CI, 1.113-2.225; *P *= 0.010). Although impaired renal function was significantly associated with the self-care, usual activities, and anxiety/depression dimensions in the univariate analyses, these associations lost statistical significance after adjustment for covariates (Table 4).

**Table 4:** Binary multivariate logistic regression of the EQ-5D dimensions

		EQ-5D dimensions				
		
		Mobility	Self-care	Usual activity	Pain/discomfort	Anxiety/depression
Age	< 40	Reference	Reference	Reference	Reference	Reference
	40-60	2.796 (1.196-4.354)^#^	9.205 (2.148-39.441)**	3.137 (1.986-4.955)^#^	1.601 (1.347-1.903)^#^	1.347 (1.118-1.623)**
	≥ 60	7.230 (4.462-11.716)^#^	13.686 (3.095-60.511)**	5.788 (3.529-9.494)^#^	3.254 (2.537-4.174)^#^	1.603 (1.243-2.068)**
Sex	male	0.452 (0.354-0.578)^#^	-	0.565 (0.440-0.727)**	0.543 (0.464-0.635)^#^	0.461 (0.364-0.584)^#^
eGFR	≥ 90.0	Reference	--	--	Reference	--
	60.0-89.9	1.863 (1.053-3.296)*			1.240 (0.982-1.565)	
	30.0-59.9	2.192 (1.178-4.077)*			1.574 (1.113-2.225)*	
Diabetes mellitus		--	--	--	--	--
Hypertension		1.412 (1.419-1.735)*	--	--	1.225 (1.045-1.435)*	--
Metabolic syndrome		--	--	--	--	--
Ischemic heart disease		2.403 (1.523-3.791)^#^	--	1.968 (1.245-3.110)**	2.515 (1.558-4.058)^#^	1.987 (1.316-2.999)**
Cerebrovascular disease		3.056 (1.886-4.953)^#^	5.326 (3.091-9.179)^#^	3.018 (1.907-4.777)^#^	1.578 (0.987-2.524)	1.833 (1.188-2.827)**
Anemia		--	--	1.385 (1.029-1.864) *	-	-
Alcohol intake		--	0.518 (0.306-0.877)*	--	--	--
Smoking		--	-	-	--	1.329 (1.060-1.667)*
Physical inactivity		1.246 (0.978-1.589)	1.978 (1.098-3.564)*	1.435 (1.098-1.875)**	-	1.158 (0.978-1.372)
Marital status	without spouse	--	--	--	--	--
Occupation	white collar	Reference	Reference	Reference	Reference	Reference
	blue collar	1.401 (0.987-1.988)	0.935 (0.436-2.005)	1.336 (0.920-1.939)	1.540 (1.272-1.863)^#^	1.194 (0.960-1.486)
	other^†^	1.403 (0.985-1.998)	1.318 (0.619-2.808)	1.355 (0.926-1.983)	1.342 (1.108-1.625)**	1.213 (0.981-1.499)
	no occupation	2.293 (1.600-3.287)^#^	2.369 (1.146-4.898)*	2.148 (1.464-3.149)^#^	1.637 (1.298-2.065)^#^	1.581 (1.234-2.026)**
Education	≥ university	Reference	Reference	Reference	Reference	Reference
	upper 2ndary^‡^	1.976 (1.226-3.187)**	1.830 (0.618-5.423)	1.680 (1.026-2.751)*	1.235 (1.027-1.486)*	1.276 (1.032-1.578)*
	≤ compulsory^§^	4.347 (2.631-7.181)^#^	3.468 (1.154-10.421)*	3.221 (1.907-5.440)^#^	2.306 (1.810-2.938)^#^	1.697 (1.286-2.240)^#^
Income	highest quartile	Reference	Reference	Reference	Reference	Reference
	2^nd ^-3^rd ^quartile	1.215 (0.857-1.722)	1.534 (0.662-3.554)	1.563 (1.048-2.332)*	1.121 (0.938-1.339)	1.209 (0.983-1.488)
	lowest quartile	1.941 (1.367-2.758)^#^	3.005 (1.324-6.825)**	2.442 (1.632-3.654)^#^	1.590 (1.288-1.963)^#^	1.879 (1.483-2.381)^#^
Residence	rural	1.383 (1.107-1.727)**	--	1.581 (1.255-1.992)^#^	-	0.854 (0.714-1.021)
Stress	some/extreme	1.455 (1.186-1.785)^#^	-	1.496 (1.208-1.852)^#^	1.739 (1.514-1.997)^#^	3.041 (2.633-3.513)^#^
Sleep quality	insufficient	-	-	-	1.613 (1.403-1.854)^#^	-

Values shown are OR (95% CI). Cells with a dash indicate that the variable was not included in the model. Cells with two dashes indicate that the variable was included in the multivariate model but excluded after the multivariate analysis. The multivariate logistic regression analysis model was derived using the backward conditional method. † Students, soldiers and housewives; ‡ middle and high school graduates; § elementary school or no education; *P < 0.05; **P < 0.001; ^#^*P *< 0.001. eGFR, estimated glomerular filtration rate (mL/min/1.73 m^2^); OR, odd ratio; CI, confidence interval

## Discussion

This is the first population-based analysis of the impact of renal dysfunction on preference-based health utility using a generic preference-based instrument. In this cross-sectional study, we found that moderate renal dysfunction is independently associated with reduced health utility, particularly in the domains of mobility and pain/discomfort.

Until recently, increasing comorbidities, along with the progression of CKD, was thought to play an important role in reduced HRQOL in patients with renal dysfunction [12]. There are, however, conflicting data on the association between HRQOL and renal function itself, especially among patients with mild-to-moderate renal dysfunction. In the Renal Research Institute-CKD study [25], eGFR had no linear association with HRQOL, and low eGFR was not an independent determinant of reduced HRQOL. Similarly, Odden *et al. *[9] found that age-adjusted HRQOL is significantly associated with renal dysfunction but that the effect is attenuated by demographic and socioeconomic variables. However, these studies were performed using subjects who had either profound renal dysfunction [25] or a history of cardiovascular events [9], both of which are major confounders in a HRQOL analysis. Therefore, these data may not be applicable to population with mild-to-moderate renal dysfunction. On the contrary, Chin *et al. *[14] reported that an eGFR value of 45 mL/min/1.73 m^2 ^or lower is an independent determinant of impaired HRQOL in the elderly Korean population. Similarly, in a population-based study in Australia, Chow *et al. *[11] reported that an eGFR lower than 60 mL/min/1.73 m^2 ^is significantly associated with an impaired HRQOL after adjusting for comorbidities associated with CKD. In accordance with previous population-based studies, we also demonstrated that an eGFR of 30.0-59.9 mL/min/1.73 m^2 ^remains an independent predictor of impaired HRQOL after adjustment for demographic, socioeconomic and psychological factors, and major comorbidities associated with CKD. We hypothesize that the conflicting findings regarding the impact of renal function on preference based health utility are largely due to the differences in study subjects in terms of their renal function and comorbidities. Because the number and severity of comorbidities increase with the progression of CKD, it can be assumed that GFR is a more important determinant of health utility in mild-to-moderate renal dysfunction. Thus, early detection of renal dysfunction and proper therapeutic intervention are important to public health efforts aimed at improving health utility.

In this study, the dimensions of EQ-5D that were particularly affected by moderate renal dysfunction were mobility and pain/discomfort, suggesting that these two components are responsible for the reduction in health utility scores that is associated with declining renal function. Although physical inactivity or functional limitations are frequently observed even in patients with mild-to-moderate renal dysfunction and are also a modifiable risk factor for mortality [26-29], there are conflicting data regarding the impact of renal function on physical activity in these patients. Data from a community-based survey of the US adult population showed that impairment in physical function among CKD patients is related to comorbidities and old age rather than to renal function itself [26]. However, other reports have suggested that renal dysfunction is directly associated with impaired physical function in elderly persons, independent of comorbidities [14,30]. Similarly, the prevalence of frailty, of which loss of mobility is a key component, increases with decreasing renal function in elderly cohorts, independent of comorbidities. Although the reasons for the association are unclear, unmeasured confounding variables such as sarcopenia [31], inflammation [32], malnutrition, or other co-morbidities may play a role [12].

In addition to impaired mobility, we found that more than 70% of the participants with an eGFR of 30.0-59.9 mL/min/1.73 m^2 ^reported that they had some or extreme pain or discomfort, and an eGFR of 30-59.9 mL/min/1.73 m^2 ^remained an independent risk factor for self-reported problems in the pain/discomfort dimension after adjusting for covariates. Similarly, the Renal Research Institute-CKD study showed that the presence of physical pain among patients with CKD stages 3-5 was associated with lower HRQOL [25]. Unfortunately, chronic pain is often not only unrecognized, but also inadequately treated in the CKD population [33]. Therefore, regular screening for pain and the development of safe and effective treatments for chronic pain are necessary to improve HRQOL in the CKD population.

The EQ-5D is a useful preference-based measurement of HRQOL that incorporates values or utilities for health status and can be used in health-economic analyses to optimize resource allocation [34,35]. In this study, we found that age-adjusted EQ-5D utility scores in participants with moderate renal dysfunction are lower than in patients with diabetes, hypertension, asthma or chronic obstructive pulmonary disease. Despite the substantially lower health utility of these patients and the chronicity of the disease, CKD awareness is extremely low in both high- and low-income countries [36]. Indeed, the awareness rate of CKD (stage I to III) has been reported to be lower than 10%, whereas the awareness rates of diabetes and hypertension are 55.8% and 51% respectively in Korea [37]. Moreover, the World Health Organization (WHO) does not yet recognize CKD as a major chronic disease that must be prevented to reduce mortality. Even though it seems apparent that early CKD detection and proper intervention can vastly reduce healthcare expenses for end-stage renal disease, these preventive strategies are implemented less frequently than recommended, even in developed countries. In addition, according to the budget expenditure report of the Centers for Disease Control and Prevention, CKD was allotted the smallest budget considering the burden of the disease [38]. Taken together, these findings suggest that healthcare resource allocation for CKD is inadequate. Under such circumstances, the results of this study provide evidence that moderate renal dysfunction may be worthy of a proportionate allotment of the available healthcare resources.

This cross-sectional study has several limitations that needed to be addressed. First, the present data showed skewed distributions of gender and eGFR groups. In this study, the proportion of the subjects in the normal renal function (eGFR ≥ 90.0 mL/min/1.73 m^2^) group was lower than that of the mildly decreased renal function group (eGFR 60.0-89.9 mL/min/1.73 m^2^). In addition, the proportion of women was higher compared with men, especially in the stage III CKD group compared with other population-based studies [39]. Although these deviant distributions may be partly explained by the inaccuracy of the MDRD equations in Asian populations [40], and an incorrect coefficient factor for female gender, which underestimates true GFR [41], the possibility of potential selection bias cannot be ruled out in this study. Second, the possible confounding effect of age which is strongly associated with both CKD and health utility could also affect the results. Furthermore, the associations we observed were only inferred from this analysis, and unmeasured residual confounding should be considered in when interpreting our results. Third, the method for serum creatinine measurement was not calibrated to be traceable to IDMS. Thus, there is the possibility of under-estimating the GFR in participants with GFR over 60 mL/min/1.73 m^2 ^[42]. Finally, no longitudinal data were available on the associations between health utility and mortality or progression to end stage renal disease among CKD participants. The precise

reason why renal impairment contributes to decreased health utility was not investigated in this cross-sectional analysis, and the interventions that could positively affect CKD patients' health utility remain unknown.

## Conclusions

In this study, moderate renal dysfunction was independently associated with impaired health utility in a Korean adult population, even though age had substantial influence on the association. Reduced mobility and increased pain or discomfort were the two dimensions significantly that were affected by moderate renal dysfunction. In addition, subjects with moderate renal dysfunction showed lower age-adjusted health preference scores than those subjects with major chronic diseases including diabetes and hypertension. These results indicate that more careful assessment of preference-based utility and proper healthcare resource allocation are required for patients with moderate renal dysfunction to improve clinical outcomes.

## Competing interests

The authors declare that they have no competing interests.

## Authors' contributions

All authors contributed extensively to the work presented in this paper at all stage. HL, YSK and DKK conceived the design of this research and wrote the manuscript. DKK supervised this project. YJO and JPL assembled the input data. MK and HK performed the statistical analyses. KHO and KWJ interpreted the data analyses. SK provided critical revision of the manuscript. HJC, CSL and SK gave conceptual advice and commented on the manuscript. All authors read and approved the final manuscript.

## Pre-publication history

The pre-publication history for this paper can be accessed here:

http://www.biomedcentral.com/1471-2369/13/19/prepub
